# Gastric and colonic metastases of malignant melanoma diagnosed during endoscopic evaluation of symptomatic anemia presenting as angina: a case report

**DOI:** 10.3389/fmed.2023.1268973

**Published:** 2023-11-02

**Authors:** Manuel A. Amaris, Henrique E. Kallas, David H. Gonzalo, Frank A. Orlando

**Affiliations:** ^1^Division of Gastroenterology, Hepatology and Nutrition, Department of Internal Medicine, College of Medicine, University of Florida, Gainesville, FL, United States; ^2^Division of Geriatrics, Department of Medicine, College of Medicine, University of Florida, Gainesville, FL, United States; ^3^Department of Pathology, Immunology and Laboratory Medicine, College of Medicine, University of Florida, Gainesville, FL, United States; ^4^Department of Community Health and Family Medicine, College of Medicine, University of Florida, Gainesville, FL, United States

**Keywords:** melanoma, gastrointestinal, metastasis, gastric, colonic, iron deficiency anemia, multifocal, BRAF

## Abstract

A 72-year-old man visited cardiology for exertional chest pain, lightheadedness, and fatigue. Six years prior, he was surgically treated for cutaneous malignant melanoma of the lower back. After a negative cardiac work-up, primary care diagnosed severe iron deficiency anemia. Emergent upper and lower gastrointestinal (GI) endoscopy revealed simultaneous melanoma metastases to the stomach and colon with discrete macroscopic features. Metastatic disease, including brain, lung, and bone, was discovered on imaging. Treatment included immunotherapy with nivolumab and stereotactic radiosurgery of the brain metastases, and our patient has remained in continued remission even after 2 years. Melanoma with GI tract (GIT) metastasis has a poor prognosis and rarely presents symptomatically or with synchronous gastric and colonic lesions. This case illustrates the importance of early primary care involvement to expedite work-up for multifocal GI metastases in patients with a remote melanoma history presenting with symptoms related to iron deficiency anemia (IDA).

## Introduction

Cutaneous melanoma is among the most common malignancies to metastasize to the GIT (1), despite the GIT being the second least common location for melanoma metastasis (2). They most commonly metastasize to the GIT from extremities (15–57%), followed by the trunk (13–54%) and head/neck (5–33%) (3). GIT metastases are usually asymptomatic, with only 1–5% of melanoma patients having clinically apparent GI involvement (3) and up to 43.5% having GIT metastasis at autopsy (4). It can be difficult to determine if a GIT melanoma is metastatic or primary when there is no synchronous cutaneous primary. Metastatic GIT melanoma can present decades later as recurrence (4, 5) or from a spontaneously regressed primary (6, 7).

Primary GI-mucosal melanomas are typically esophageal or anorectal (8), not gastric or colonic (9), and are usually aggressive with a worse prognosis than melanoma metastatic to the GIT (4). The small bowel is the most common location for GIT metastasis (2, 3, 6, 10), and the stomach or colon is less common (2, 10). A retrospective cohort describing 55% of secondary GIT melanomas as “multifocal” probably noted a greater frequency in the upper GIT because it included small bowel (1). Here, we describe a rare case of melanoma presenting with angina from symptomatic anemia and with simultaneous gastric and colonic metastases.

## Case presentation

A 72-year-old man with atrial fibrillation on apixaban presented in September 2021 (Table 1) to his cardiologist with exertional chest pressure, postural lightheadedness, and fatigue for weeks. An echocardiogram and stress test were unrevealing. Cardiac catheterization showed mild, non-obstructive coronary artery disease. Cardiology lab results showed severe microcytic anemia (hemoglobin 7.0 G/DL) and primary care lab results showed iron deficiency (ferritin 7.6 NG/ML), although there was no overt GI hemorrhage. Apixaban was held, and an emergent upper and lower endoscopy was ordered (11).

**Table 1:** Patient care timeline.

**Date**	**Event**
12/2015	Wide local excision and sentinel lymph node biopsy for cutaneous melanoma of lower back
9/2021	Presents to cardiology with angina
10/2021	Primary care diagnoses iron deficiency anemia; endoscopies and imaging diagnose metastatic melanoma; starts nivolumab immunotherapy; undergoes stereotactic radiosurgery for two brain metastases
1/2022	First monitoring PET-CT shows cancer remission at cycle 3
6/2022	Vitiligo develops at cycle 9
8/2022	Guttate psoriasis develops at cycle 11 and is treated
9/2022	Completes 12 cycles of nivolumab; oral thrush and inflammation of preexisting seborrheic keratoses develop shortly thereafter and are treated
10/22	Baseline surveillance imaging and ctDNA show continued complete remission
10/2022–7/2023	Remains in continued remission based on brain MRI, whole body PET-CT, and ctDNA every 3 months

Relevant past medical history includes a cutaneous melanoma of the lower back that was diagnosed in 2015 by punch biopsy (Clark Level IV, Breslow Depth 2.25 mm, mitotic index approximately 1 mitosis/mm^2^, no significant tumor regression, epidermal ulceration, microvascular invasion, or satellite micro-metastases), which was at clinical stage IIA (T3a N0 M0) (Supplementary Figure S1). A wide local excision had negative margins, and a sentinel lymph node biopsy was negative. The pathological stage was also IIA and further staging work-up was therefore not performed based on the early stage of this typical superficial spreader. Adjuvant therapies were experimental at the time and not prescribed. Bi-annual skin and periodic ophthalmologic exams remained unremarkable. Screening colonoscopies were up to date (12), and a 25-mm tubular adenoma had been removed 4 years prior.

**Figure 1:**
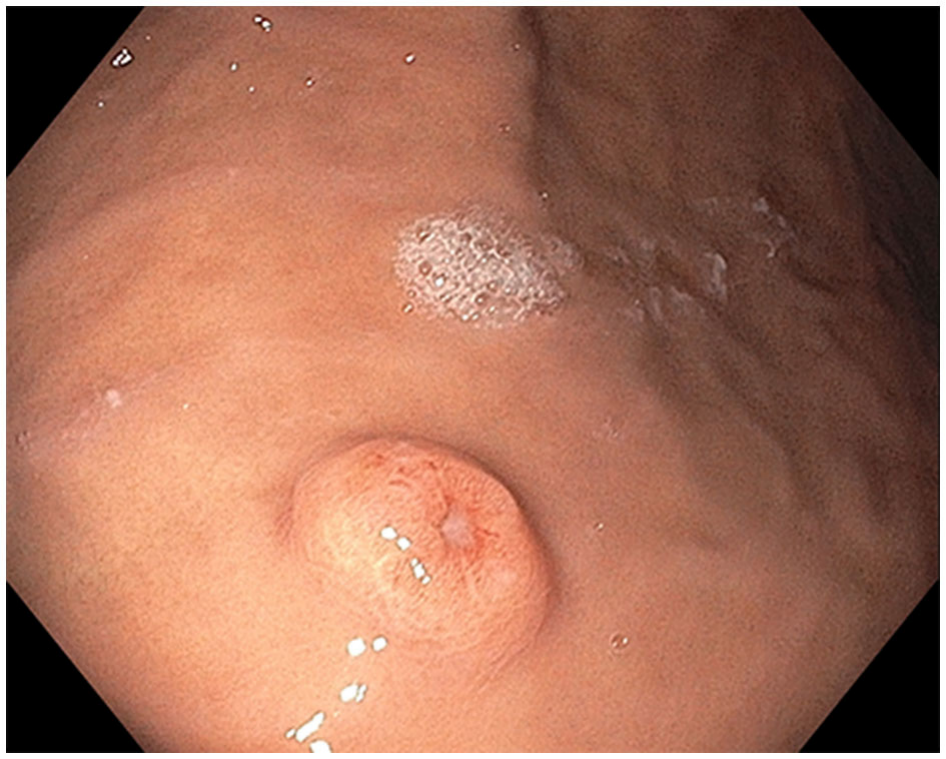
Stomach polyp: Single non-melanotic, 12 mm in diameter and 2.5 mm in height, sessile umbilicated polyp (equivalent to a Paris-*1s* classification of colonic polyps) found on the gastric body.

**Figure 2:**
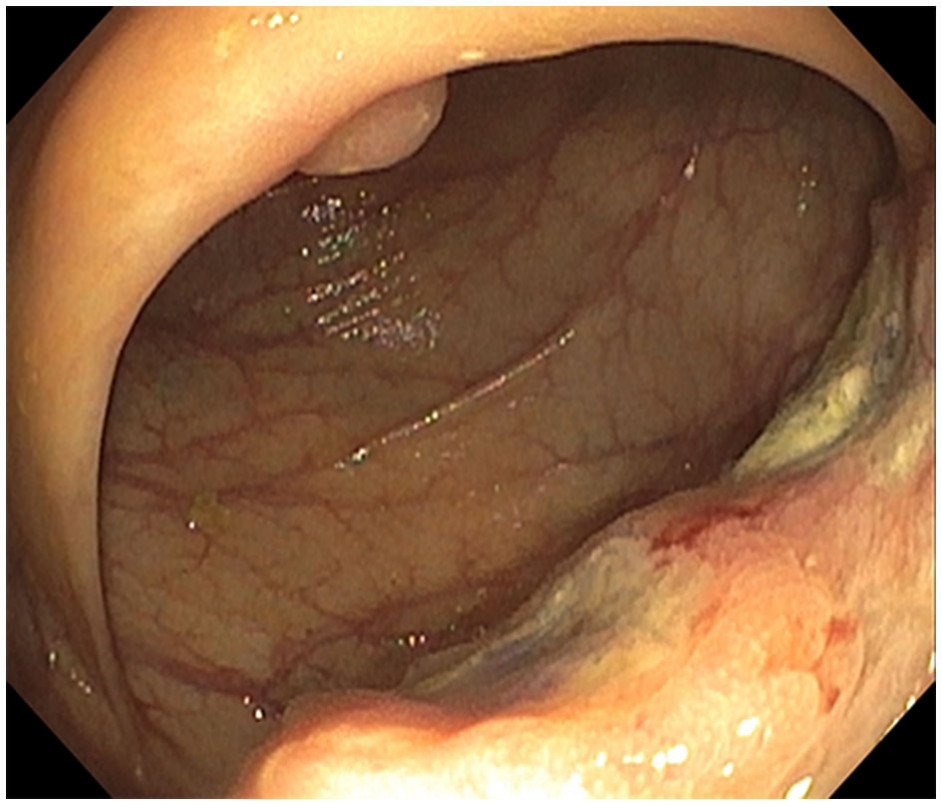
Ascending colon mass: infiltrative and ulcerated non-melanotic, partially obstructing 4-cm mass involving one-half of the lumen circumferences.

**Figure 3:**
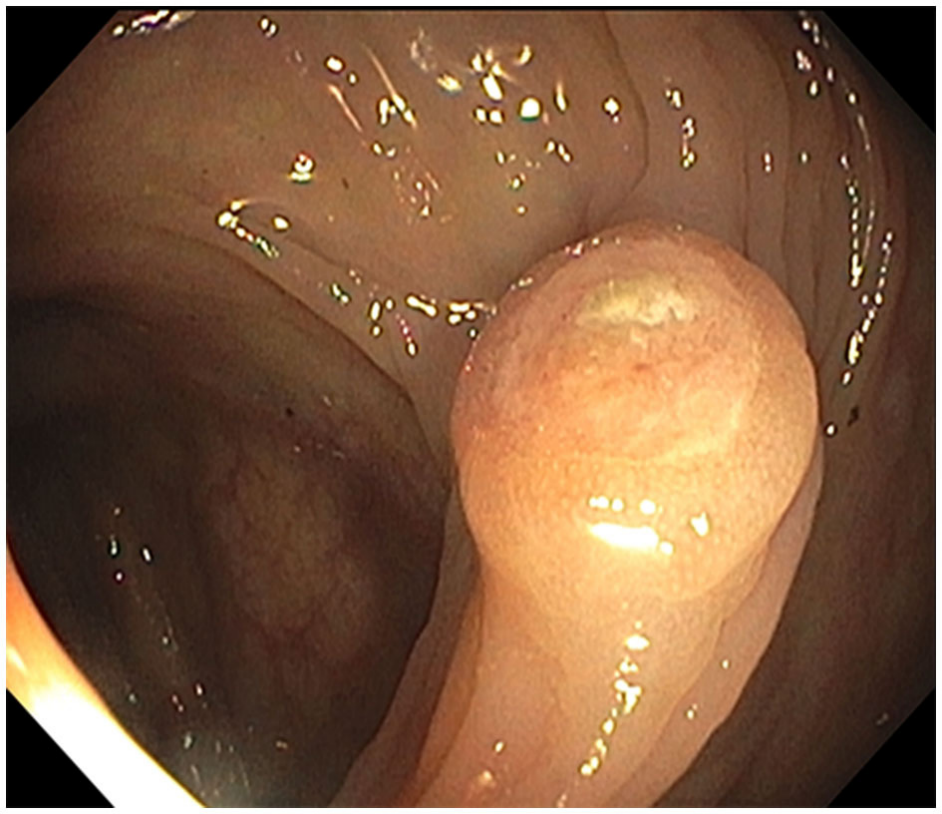
Hepatic flexure polyp: endoscopic appearance of the non-melanotic, 12 mm in diameter and 3 mm in height, sessile umbilicated polyp (Paris-*1s* classification of colonic polyps).

In October 2021, esophagogastroduodenoscopy and colonoscopy identified a gastric body polyp (Figure 1, Supplementary Figure S2), a partially obstructing ascending colon mass (Figure 2), and hepatic flexure polyp (Figure 3, Supplementary Figure S3). All three biopsies diagnosed metastatic melanoma (Supplementary Figures S4–S6). PET-CT found lung and right humeral head metastases, but none of the GI metastases were detectable. Abdomen/pelvis CT with contrast was non-contributory, but a chest CT demonstrated bilateral solid pulmonary nodules up to 2.5 cm, a 2.1 cm right humeral head lytic lesion, and a few left supraclavicular lymph nodes up to 1.0 cm, corresponding to PET-CT. A brain MRI showed left caudate and cerebellar metastases. Serum lactate dehydrogenase (LDH) was normal, but GI metastases harbored *BRAF* V600E, *PTEN*, and *TERT* mutations. The pathological stage was IV [rTX, N2b, M1d(0)] based on the American Joint Committee on Cancer (AJCC) 8th Edition staging system (13).

The patient's symptomatic anemia was successfully treated with two iron infusions. He underwent stereotactic radiosurgery for the brain metastases without complications. Twelve cycles of immunotherapy with nivolumab (480 mg monthly infused over 30 min) were completed in September 2022. Nivolumab-induced vitiligo developed at cycle 9 and guttate psoriasis on the chest, arms, and legs at cycle 11, the latter of which resolved with 0.1% triamcinolone cream twice daily. Shortly after cycle 12, he developed oral candidiasis, and preexisting seborrheic keratoses became inflamed, both thought to be nivolumab-induced. The former was treated with oral nystatin, and the latter with either a topical steroid or cryotherapy. Immunotherapy response was monitored with monthly circulating tumor DNA (ctDNA) levels and PET-CTs every 3 months. The patient went into remission after 3 months of immunotherapy and remains in continued remission as of July 2023. His surveillance has included brain MRI and whole-body PET-CT at baseline and every 3 months, along with ctDNA testing that was reduced from monthly after 6 months of disease-free surveillance.

## Discussion

Our patient's cutaneous melanoma 6 years prior is congruent with the interim average and median reported times from primary cutaneous melanoma treatment to find a GI metastasis, or 3.65 years (10) and 10 years (14), respectively. However, the locations of his metastases were very unusual. We found no reports of simultaneous upper and lower GIT lesions in metastatic GIT cases and only two reports in primary GIT cases: gastric/ileocecal (15) and esophageal/ileal (16). While our patient did have several risk factors for multiple primary GI-mucosal melanomas, including older age at diagnosis of first melanoma (66 years old), male sex, and white race (17), these were unlikely to occur in the stomach or colon (8, 9), and proposed criteria for other intestinal locations require a solitary lesion (6).

Prompt diagnosis is important in symptomatic GIT metastasis because of life-threatening complications (18), which were prevented in this case. In addition to IDA, patients can present with abdominal pain, dysphagia, small bowel obstruction, and/or perforation (1, 4, 19). Endoscopy is superior to radiography for diagnosing GIT melanoma and its complications (19), and capsule endoscopy may be necessary (5). GIT melanoma appears as either pigmented or amelanotic ulcerated polypoid lesions (1, 4, 19).

GIT metastasis is a poor prognostic marker (5-year survival 14%, median survival 12.5 months) (3), and autopsy often reveals multiple organ metastases (95%) (2). GI metastases are not typically the cause of death, which is usually respiratory failure from lung metastasis (2). LDH elevation is a negative predictor of survival in the AJCC staging system (13) but is not melanoma-specific.

*BRAF, PTEN*, and *TERT* mutations were present in his melanoma metastases. Half of cutaneous melanomas have *BRAF* mutations, and the most common V600E mutation is associated with decreased overall survival in advanced melanoma (20). *BRAF* mutation testing is required for resectable or unresectable melanoma stage III or stage IV, highly recommended for stage IIC high-risk resected disease, and not recommended for stage I or stages IIA–IIB (21). His overall survival was decreased with *PTEN* and *TERT* mutations, both of which often coexist with *BRAF* mutations (22, 23). Interestingly, however, the vitiligo he experienced during his immunotherapy has been associated with progression-free and overall survival that is significantly increased (24).

## Conclusion

Melanoma often progresses to metastatic disease but can be challenging to quickly diagnose in the absence of a cutaneous lesion. GIT metastasis has a particularly poor prognosis. Synchronous gastric and colonic melanomas are extremely rare but important to consider in patients with IDA and a remote history of melanoma because of their poor prognosis and potential to cause an abdominal emergency. The patient's metastatic melanoma presented as angina, showing the importance of early primary care involvement to rapidly diagnose the cause of symptomatic IDA. The expedited upper and lower endoscopy and multidisciplinary involvement prevented abdominal complications and began timely treatment. Our patient has remained in continued remission even after 2 years.

## Data availability statement

The original contributions presented in the study are included in the article/Supplementary material, further inquiries can be directed to the corresponding author.

## Ethics statement

Ethical approval was not required for the studies involving humans because we contacted our Institutional Review Board, and their approval is not required for case reports. We contacted our ethics committee, and their approval is also not required for de-identified case reports with all protected health information is removed and with written consent from the patient who has reviewed the report. The studies were conducted in accordance with the local legislation and institutional requirements. The participants provided their written informed consent to participate in this study. Written informed consent was obtained from the individual(s) for the publication of any potentially identifiable images or data included in this article.

## Author contributions

FO: Conceptualization, Funding acquisition, Investigation, Project administration, Software, Writing—original draft, Writing—review & editing. MA: Conceptualization, Data curation, Funding acquisition, Investigation, Writing—review & editing. HK: Data curation, Funding acquisition, Investigation, Methodology, Writing—original draft, Writing—review & editing. DG: Data curation, Funding acquisition, Investigation, Software, Writing—review & editing.

## Supplementary material

Supplementary Figure S1 **(A)** Skin, mid-right back, punch biopsy: non-encapsulated well circumscribed tumoral nodule (H&E, original magnification 40X). **(B)** Malignant melanoma composed of a monomorphic population of epithelioid cells with melanin pigment (H&E, original magnification 400X).

Supplementary Figure S2 **(A, B)** Stomach polyp: Additional close-up, endoscopic views of single non-melanotic, 12 mm in diameter and 2.5 mm in height, sessile umbilicated polyp (equivalent to a Paris-*1s* classification of colonic polyps) found on the gastric body.

Supplementary Figure S3 Hepatic flexure polyp: Additional close-up view showing endoscopic appearance of the 12 mm in diameter and 3 mm in height non-melanotic sessile umbilicated polyp (Paris-*1s* classification of colonic polyps).

Supplementary Figure S4 Stomach polyp: Melanoma cells undermining gastric epithelium (H&E, original magnification 200X).

Supplementary Figure S5 Ascending colon mass: Tumoral cells (arrows) flanked by colonic crypts (H&E, original magnification 200X).

Supplementary Figure S6 D. Hepatic flexure polyp: Melanoma cells stain strongly for melanocytic marker SOX-10 (SOX-10 immunohistochemical stain, original magnification 20X).
