# Supplementary figures and images for: Gastric and colonic metastases of malignant melanoma diagnosed during endoscopic evaluation of symptomatic anemia presenting as angina: a case report
Source: Front Med (Lausanne). 2023 Nov 2;10:1268973. doi: 10.3389/fmed.2023.1268973 (PMC10652385; doi:10.3389/fmed.2023.1268973)

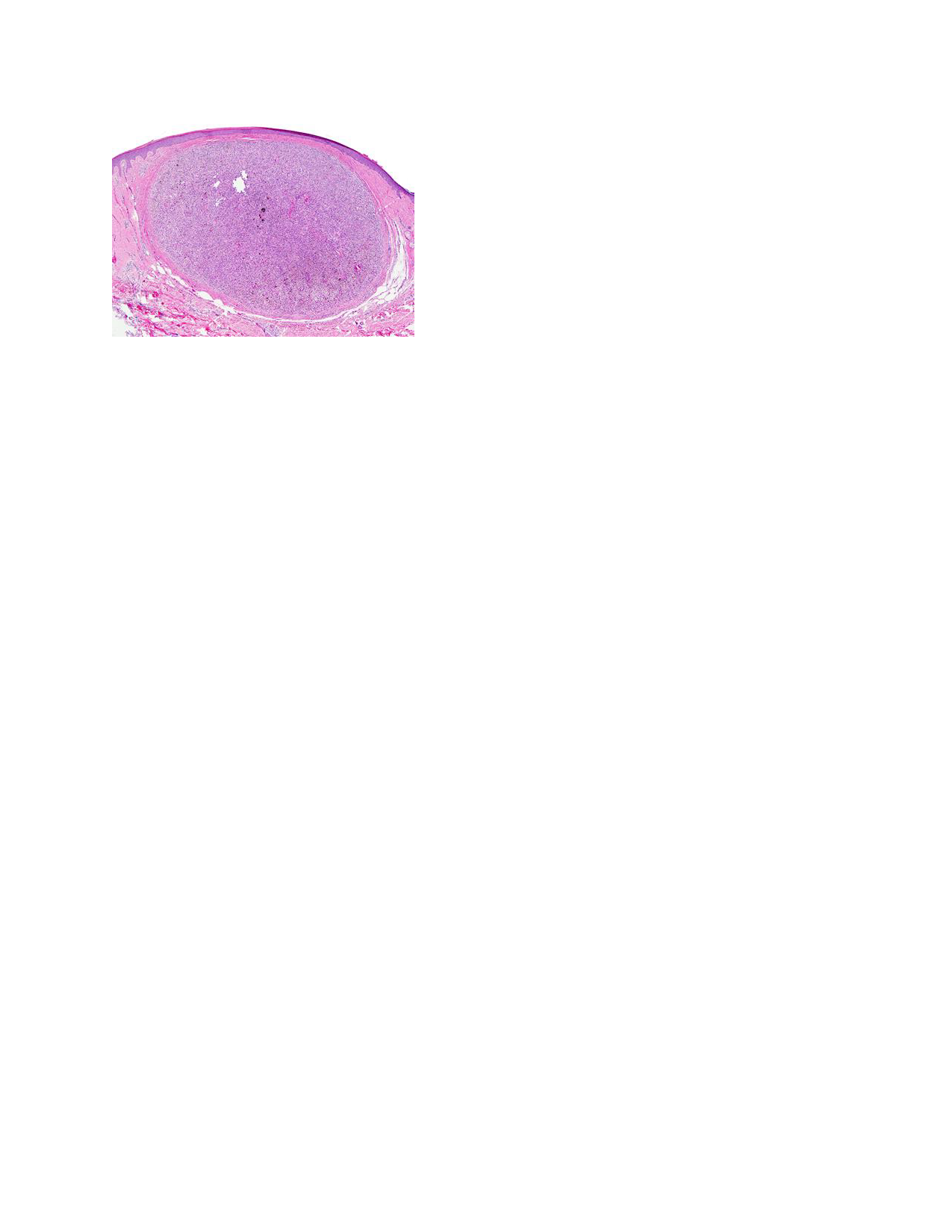

Supplement: Supplementary Figure S1 — (A) Skin, mid-right back, punch biopsy: non-encapsulated well circumscribed tumoral nodule (H&E, original magnification 40X). (B) Malignant melanoma composed of a monomorphic population of epithelioid cells with melanin pigment (H&E, original magnification 400X). [file Image_1.TIFF]

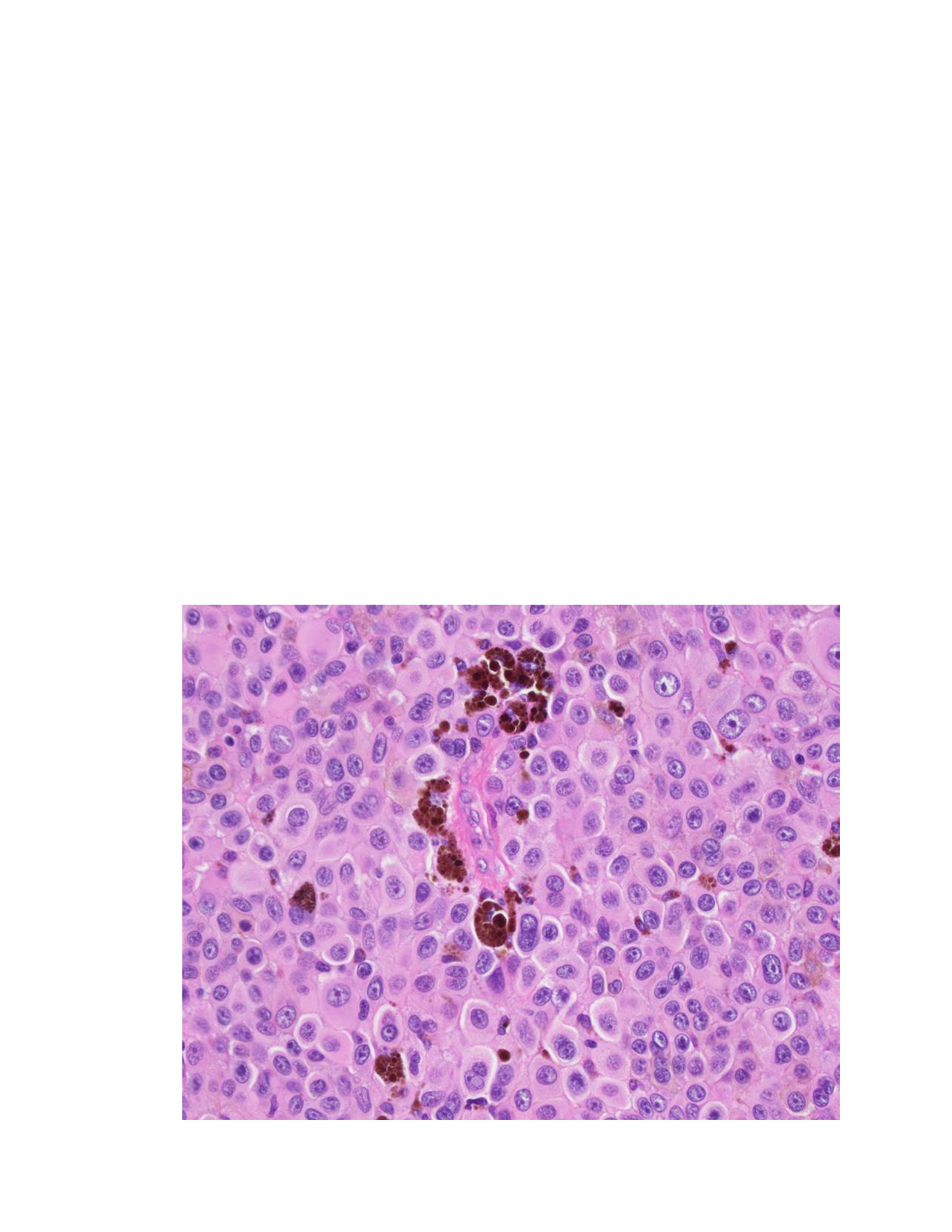

Supplement: Supplementary Figure S2 — (A, B) Stomach polyp: Additional close-up, endoscopic views of single non-melanotic, 12 mm in diameter and 2.5 mm in height, sessile umbilicated polyp (equivalent to a Paris-1s classification of colonic polyps) found on the gastric body. [file Image_2.TIF]

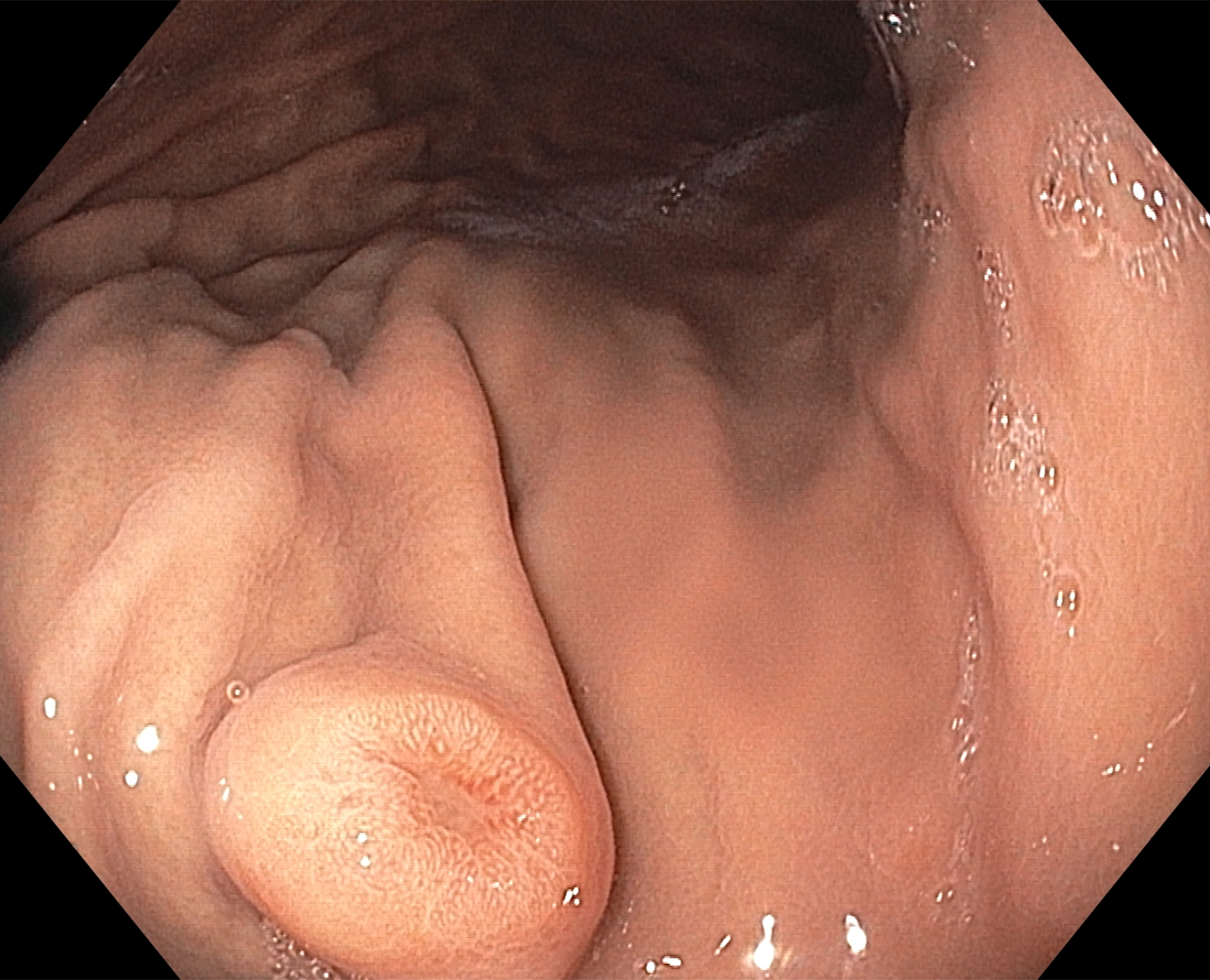

Supplement: Supplementary Figure S3 — Hepatic flexure polyp: Additional close-up view showing endoscopic appearance of the 12 mm in diameter and 3 mm in height non-melanotic sessile umbilicated polyp (Paris-1s classification of colonic polyps). [file Image_3.TIF]

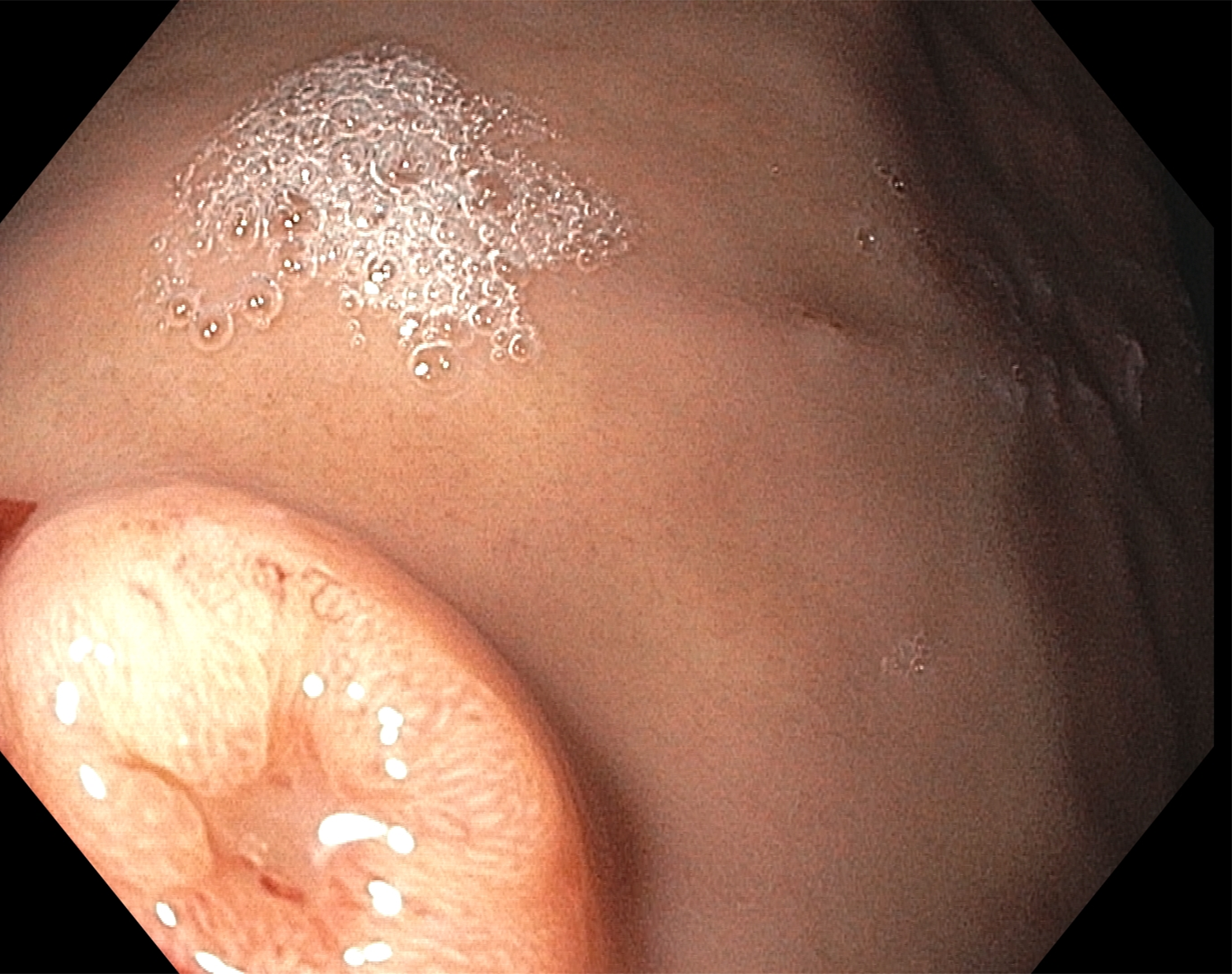

Supplement: Supplementary Figure S4 — Stomach polyp: Melanoma cells undermining gastric epithelium (H&E, original magnification 200X). [file Image_4.TIF]

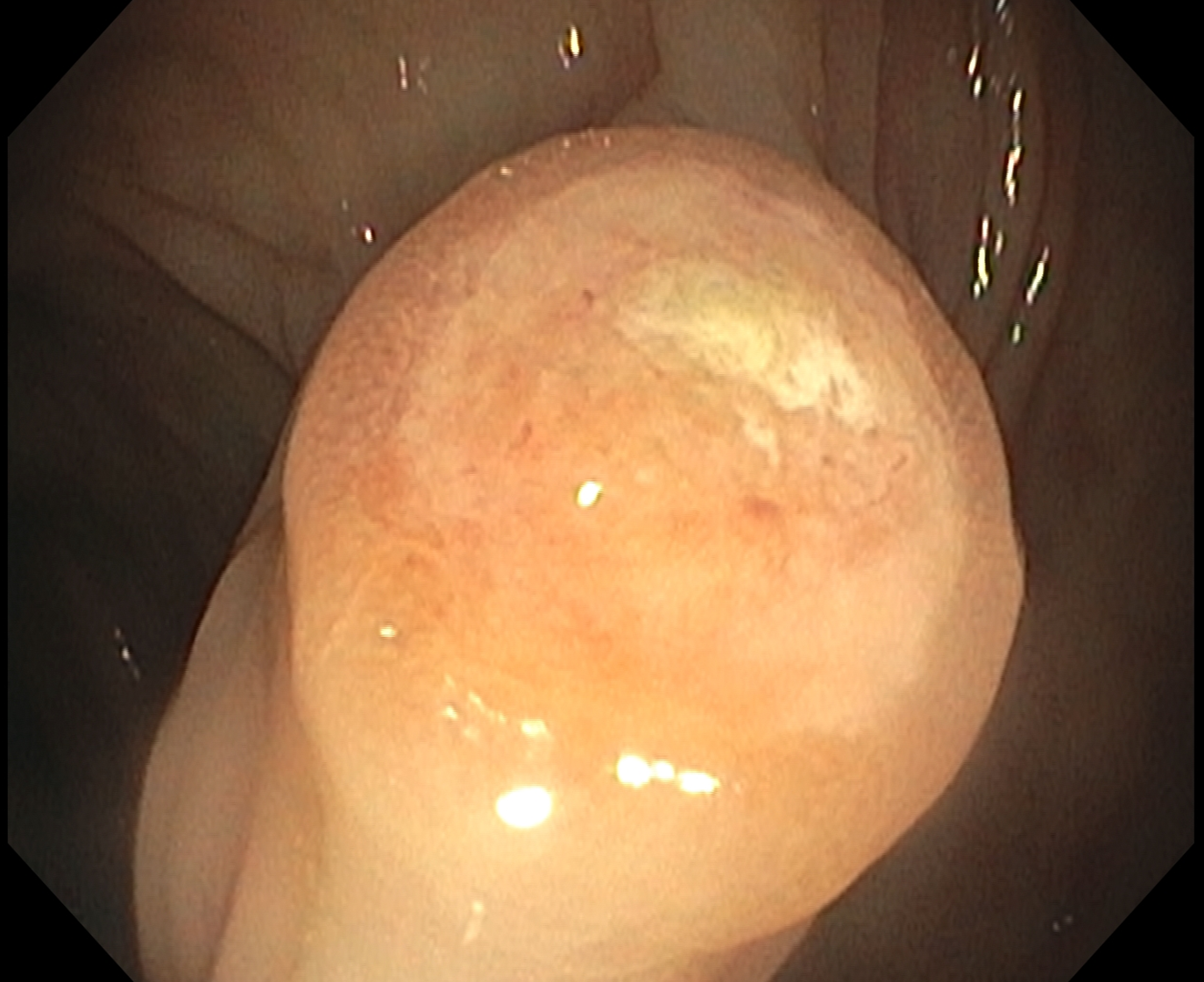

Supplement: Supplementary Figure S5 — Ascending colon mass: Tumoral cells (arrows) flanked by colonic crypts (H&E, original magnification 200X). [file Image_5.TIF]

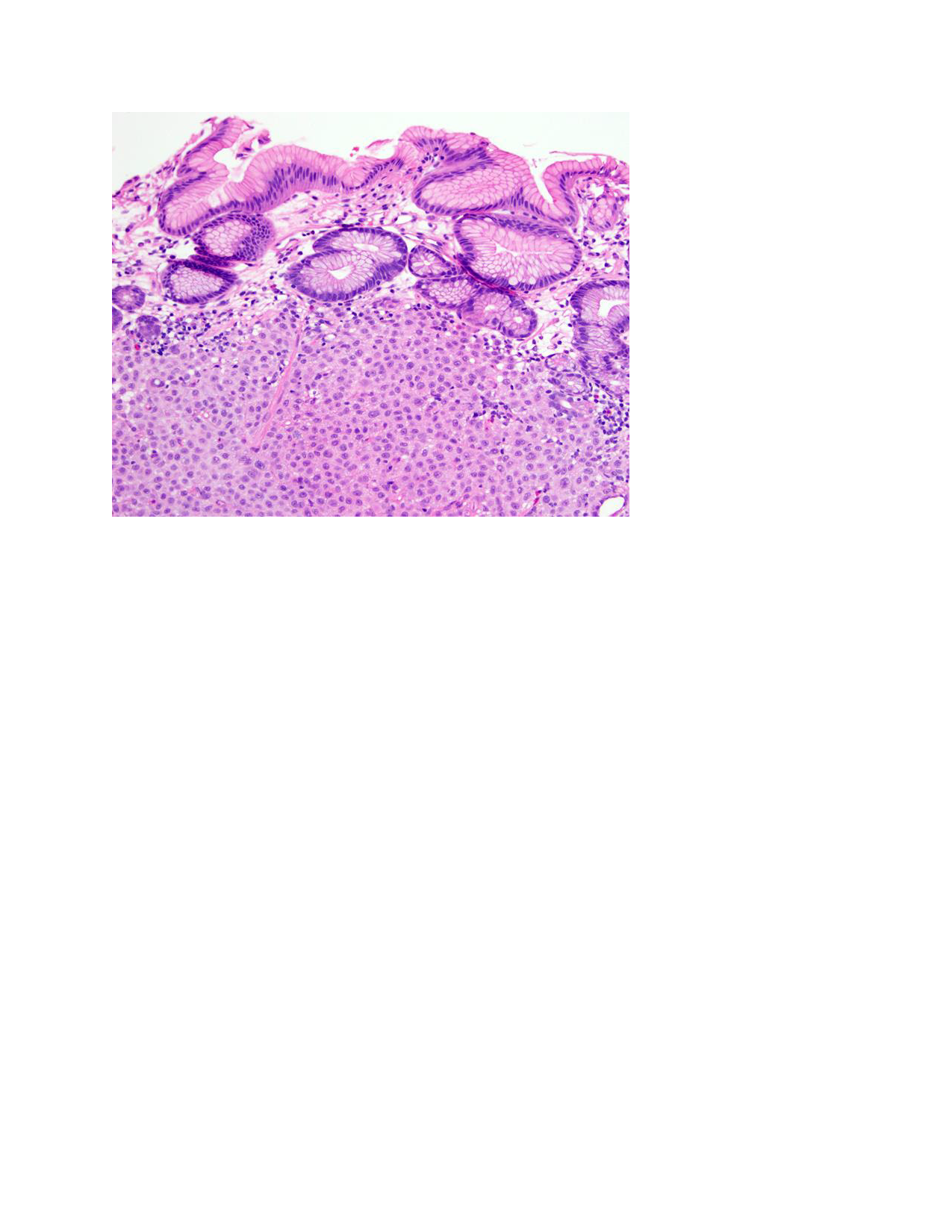

Supplement: Supplementary Figure S6 — D. Hepatic flexure polyp: Melanoma cells stain strongly for melanocytic marker SOX-10 (SOX-10 immunohistochemical stain, original magnification 20X). [file Image_6.TIF]

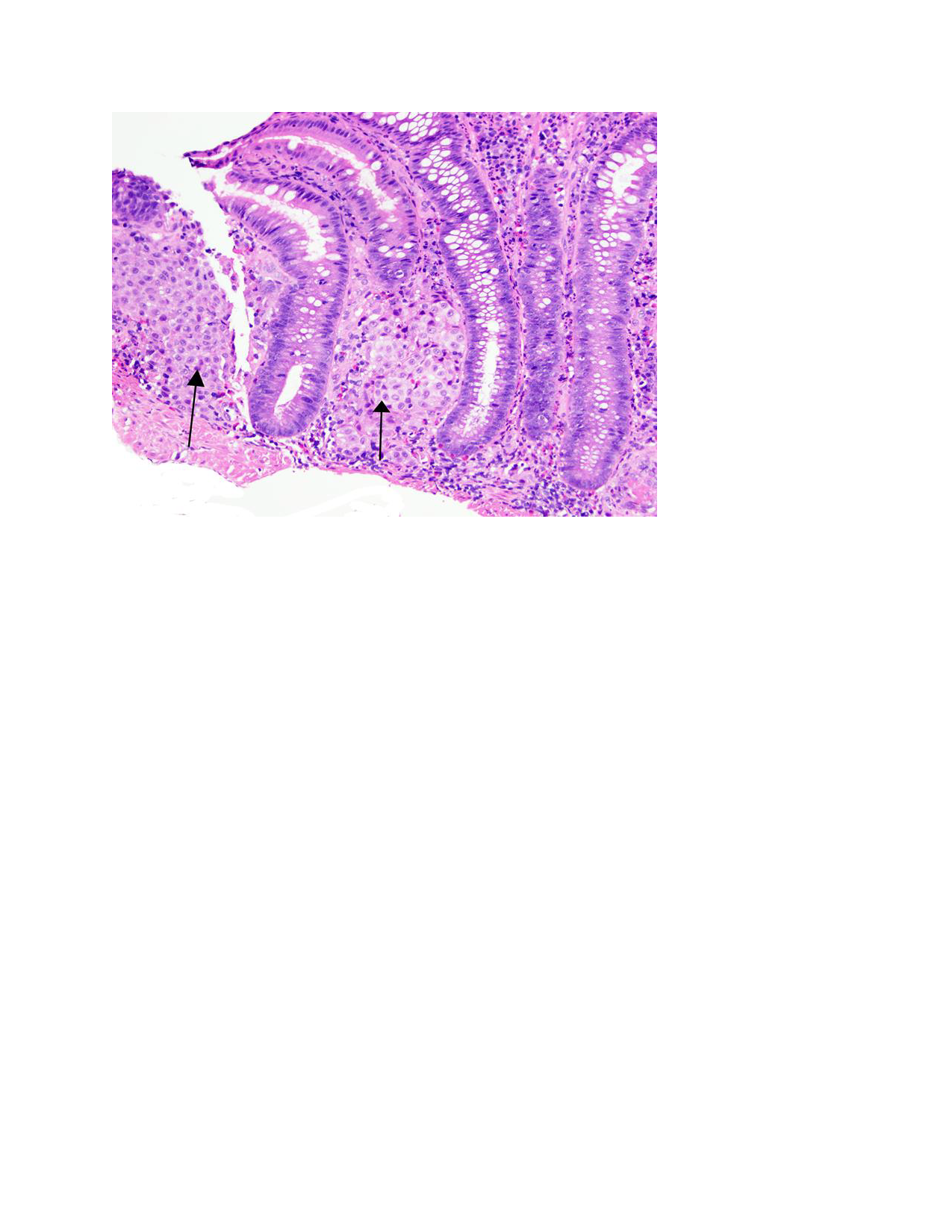

Supplement: Supplementary file 7 [file Image_7.TIF]

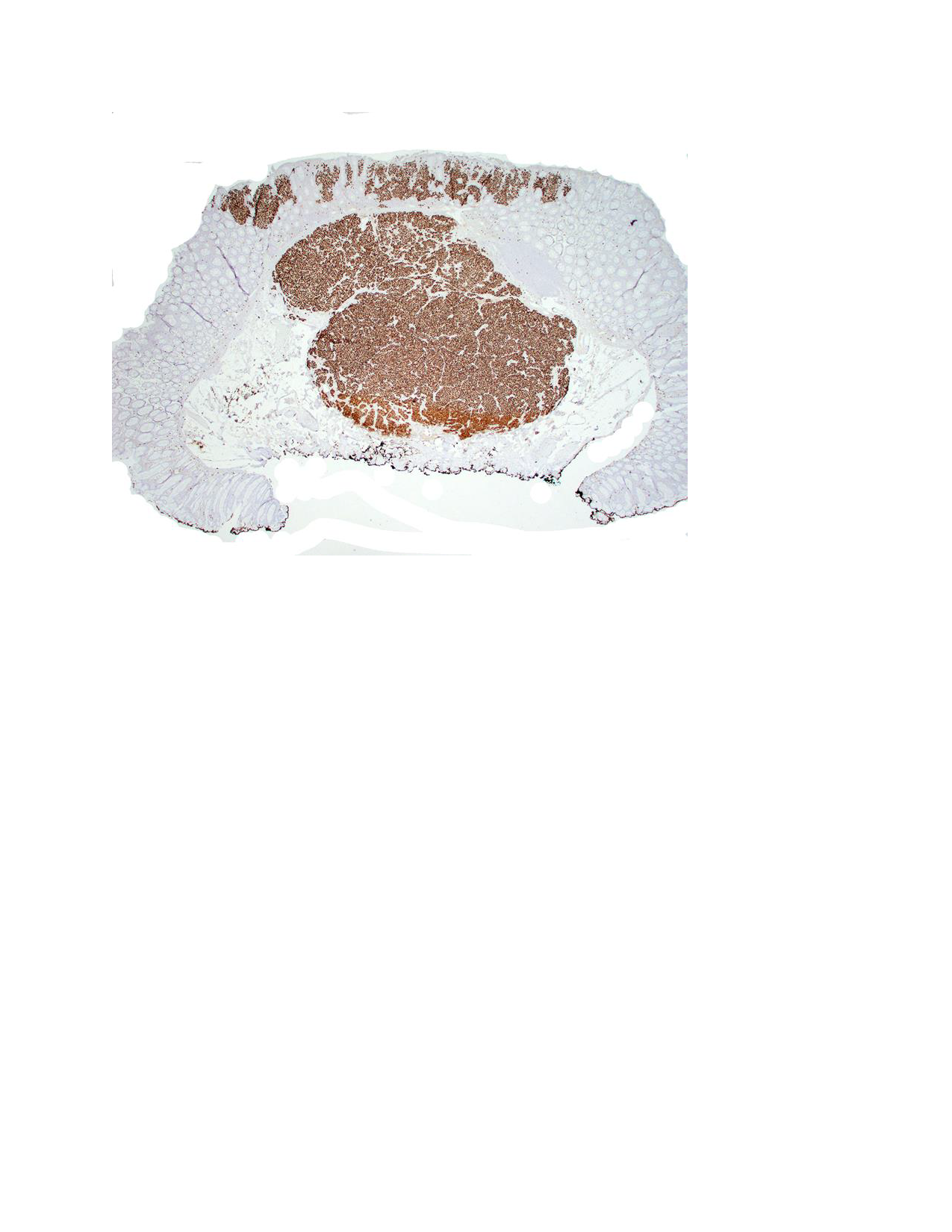

Supplement: Supplementary file 8 [file Image_8.TIF]
